# A novel DDAH-1 inhibitor improved cardiovascular function in a short-term anesthetized rat model of sepsis

**DOI:** 10.1186/cc10399

**Published:** 2011-10-27

**Authors:** Z Wang, V Taylor, R Stidwill, J Leiper, M Singer

**Affiliations:** 1 Bloomsbury Institute of Intensive Care Medicine, Department of Medicine, University College London, UK

## Introduction

Excessive NOS activity and NO overproduction are believed to play an important role in sepsis-induced macrocirculatory and microcirculatory dysfunction. Asymmetric dimethylarginine (ADMA), an endogenous inhibitor of NO synthesis, is extensively metabolised by dimethylarginine dimethylaminohydrolase (DDAH). The DDAH-1 isoform is present in vascular smooth muscle so its inhibition should theoretically reverse sepsis-induced hypotension. We thus investigated the dose-dependent cardiovascular effects of a novel DDAH-1 competitive inhibitor, L-257, in experimental sepsis.

## Methods

Anaesthetised, spontaneously breathing male Wistar rats (body weight 270 to 330 g) had their left carotid artery and right internal jugular vein cannulated for arterial pressure monitoring and fluid infusion, respectively. Then 40 mg/kg *Klebsiella pneumoniae *lipopolysaccharide was administered intravenously over 30 minutes followed by fluid resuscitation at a rate of 10 ml/kg/hour thereafter. When the mean arterial pressure fell over 20% below baseline, groups (*n *= 6) were randomized to receive a bolus dose of L-257 of 0 (control), 3, 30 or 300 mg/kg. Animals were sacrificed 2 hours later with prior measurement of gastrocnemius muscle microcirculatory perfusion and with collection of plasma samples for biochemistry, arginine, ADMA and nitrate/nitrite measurements.

## Results

**Figure 1:**
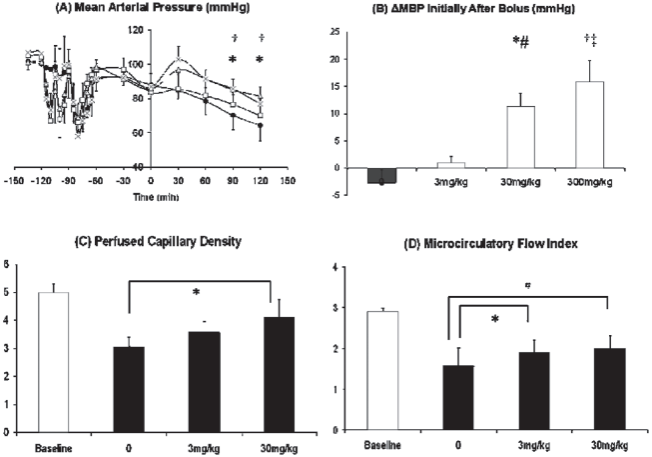
**(a) The effect of bolus doses of L-257 at 0 mg/kg, 3 mg/kg, 30 mg/kg and 300 mg/kg on the mean arterial pressure in the short-term organ function study on anaesthetized Wistar rats (*n *= 6 in the each group)**. The bolus L-257 was given at 0 minutes for 10-minute infusion. Animals treated with 300 mg/kg L-257, x; animals treated with 30 mg/kg L-257, open triangle; animals treated with 3 mg/kg, open square; control animals, solid circle. **(b) **The initial change of mean arterial pressure 30 minutes after bolus injections among the four groups. *P < 0.05, 30 mg/kg vs. control; ^#^*P *< 0.05, 30 mg/kg vs. 3 mg/kg; ^†^*P *< 0.05, 300 mg/kg vs. control; ^‡^*P *< 0.05, 300 mg/kg vs. 3 m/kg. **(c), (d) **The effects of bolus doses of L-257 at 0 mg/kg, 3 mg/kg and 30 mg/kg on perfused capillary density and microcirculatory index (*n *= 4 in each group). *P < 0.05, 30 mg/kg vs. control; ^#^*P *< 0.05, 30 mg/kg vs. 3 m/kg.

**Figure 2:**
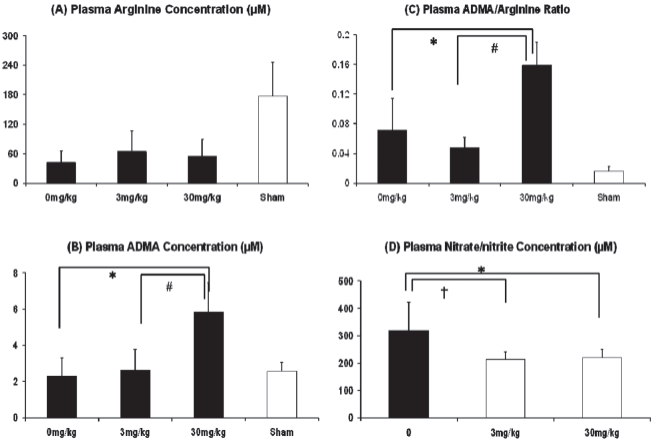
**The effect of bolus doses of L-257 at 0 mg/kg, 3 mg/kg and 30 mg/kg on (a) plasma arginine and (b) ADMA concentrations, (c) the ADMA/arginine ratio and (d) plasma nitrate/nitrite concentrations in the short-term organ function study on anaesthetized Wistar rats (*n *= 6 in each group)**. *P < 0.05, 30 mg/kg vs. control; ^#^*P *< 0.05, 30 mg/kg vs. 3 m/kg; ^†^*P *< 0.05, 3 mg/kg vs. control.

The bolus doses of L-257 were given after approximately 60 to 90 minutes post endotoxin when the mean BP fell over 20%. Arterial pressure, perfused capillary density and microcirculatory flow index were better maintained than in controls, especially at higher doses (Figure 1, *P *< 0.05). Significantly higher plasma ADMA concentrations and ADMA/arginine ratios were seen in the 30 mg/kg bolus group (Figure 2, *P *< 0.05). Plasma nitrate/nitrite levels in the treated animals were significantly lower compared with those in controls (Figure 2, *P *< 0.05).

## Conclusion

In this short-term rat model of endotoxaemia, we demonstrated protective dose-dependent effects of a novel DDAH-1 inhibitor, L-257, on cardiovascular function. This was associated with an elevation of plasma ADMA level and a resultant reduction of plasma nitrate/nitrite level.

